# A Systematic Review of the Relationship between Blood Loss and Clinical Signs

**DOI:** 10.1371/journal.pone.0057594

**Published:** 2013-03-06

**Authors:** Rodolfo Carvalho Pacagnella, João Paulo Souza, Jill Durocher, Pablo Perel, Jennifer Blum, Beverly Winikoff, Ahmet Metin Gülmezoglu

**Affiliations:** 1 Federal University of São Carlos, São Carlos, Brazil; 2 Department of Reproductive Health and Research, World Health Organization, Geneva, Switzerland; 3 Gynuity Health Projects, New York, New York, United States of America; 4 London School of Hygiene and Tropical Medicine, London, United Kingdom; Baylor College of Medicine, United States of America

## Abstract

**Introduction:**

This systematic review examines the relationship between blood loss and clinical signs and explores its use to trigger clinical interventions in the management of obstetric haemorrhage.

**Methods:**

A systematic review of the literature was carried out using a comprehensive search strategy to identify studies presenting data on the relationship of clinical signs & symptoms and blood loss. Methodological quality was assessed using the STROBE checklist and the general guidelines of MOOSE.

**Results:**

30 studies were included and five were performed in women with pregnancy-related haemorrhage (other studies were carried in non-obstetric populations). Heart rate (HR), systolic blood pressure (SBP) and shock index were the parameters most frequently studied. An association between blood loss and HR changes was observed in 22 out of 24 studies, and between blood loss and SBP was observed in 17 out of 23 studies. An association was found in all papers reporting on the relationship of shock index and blood loss. Seven studies have used Receiver Operating Characteristic Curves to determine the accuracy of clinical signs in predicting blood loss. In those studies the AUC ranged from 0.56 to 0.74 for HR, from 0.56 to 0.79 for SBP and from 0.77 to 0.84 for shock index. In some studies, HR, SBP and shock index were associated with increased mortality.

**Conclusion:**

We found a substantial variability in the relationship between blood loss and clinical signs, making it difficult to establish specific cut-off points for clinical signs that could be used as triggers for clinical interventions. However, the shock index can be an accurate indicator of compensatory changes in the cardiovascular system due to blood loss. Considering that most of the evidence included in this systematic review is derived from studies in non-obstetric populations, further research on the use of the shock index in obstetric populations is needed.

## Introduction

All women giving birth lose some amount of blood during the immediate postpartum period. In the majority of women, the postpartum blood loss is well tolerated. In some women excessive bleeding occurs and is associated with severe maternal morbidity and mortality. Postpartum haemorrhage (PPH) is one of the major causes of maternal deaths around the world and its underlying causes include uterine atony, genital tract tears and retention of placental tissue [1]. Depending on the rate of blood loss and other factors such as pre-existing anaemia, untreated PPH can lead to hypovolemic shock, multi-organ dysfunction and maternal death within 2 to 6 hours [2], [3]. Therefore, early identification and treatment of women with PPH is a key factor for maternal survival.

The diagnosis of PPH is largely based on the identification of excessive blood loss in the postpartum period. In 1990, the World Health Organization adopted the definition of PPH after vaginal delivery as the loss of 500 ml or more of blood from the genital tract after delivery of a baby. Primary PPH is usually defined as excessive blood loss that occurs within 24 hours after birth and a blood loss of 1000 ml or more is defined as severe PPH [4]. In caesarean sections, a higher threshold for diagnosing PPH (e.g. 750–1000 ml) is generally accepted.

Direct measurement is the ideal method for quantifying blood loss after birth. The majority of PPH-related maternal deaths take place in under-resourced settings and the use of direct methods (e.g. gravimetric or photometric) for quantifying blood loss in all births is not realistic [5]. Visual estimation of blood loss (VEBL) is the method most frequently used around the world in the diagnosis of PPH which is based on clinical judgment by the provider via visual estimation of blood loss. However, the use of VEBL has been associated with underestimation of the amount of blood loss [6]. Considering these limitations, other methods for estimating blood loss have been proposed (e.g. hematocrit/hemoglobin assessment) together with alternative PPH definitions (e.g. 10% drop in hematocrit/hemoglobin) [7]–[9]. Nevertheless, the added benefit of these alternative methods in comparison with VEBL seems to be minimal and their applicability in under-resourced settings is limited.

Clinical signs have been used as a surrogate for blood loss in non-obstetric populations, particularly when quantification of blood loss is not feasible (e.g. trauma and occult bleeding). More importantly, clinical signs have been used to guide fluid replacement in trauma patients with hypovolemic shock due to haemorrhage [10]. By analogy, some authors have suggested the use of clinical signs and symptoms of hypovolemia as markers of PPH [8], [11]. Signs and symptoms such as pallor, light-headedness, weakness, palpitations, tachycardia, diaphoresis, restlessness, confusion, air hunger, syncope, fatigue and oliguria have been associated with blood loss [11]. However, none of these clinical signs and symptoms has been properly correlated with different degrees of hypovolemia in obstetric populations and there has not been any systematic review to assess the relationship between blood loss and clinical signs and symptoms. Other relevant uncertainties relate to the amount of blood loss that should indicate a diagnosis of PPH and what clinical consequences of blood loss are of greatest importance in predicting consequences for women experiencing excessive blood loss.

This systematic review aims at assessing the relationship between blood loss and clinical signs and explores the potential of using such clinical findings to trigger clinical interventions in the management of PPH.

## Methods

**Figure 1:**
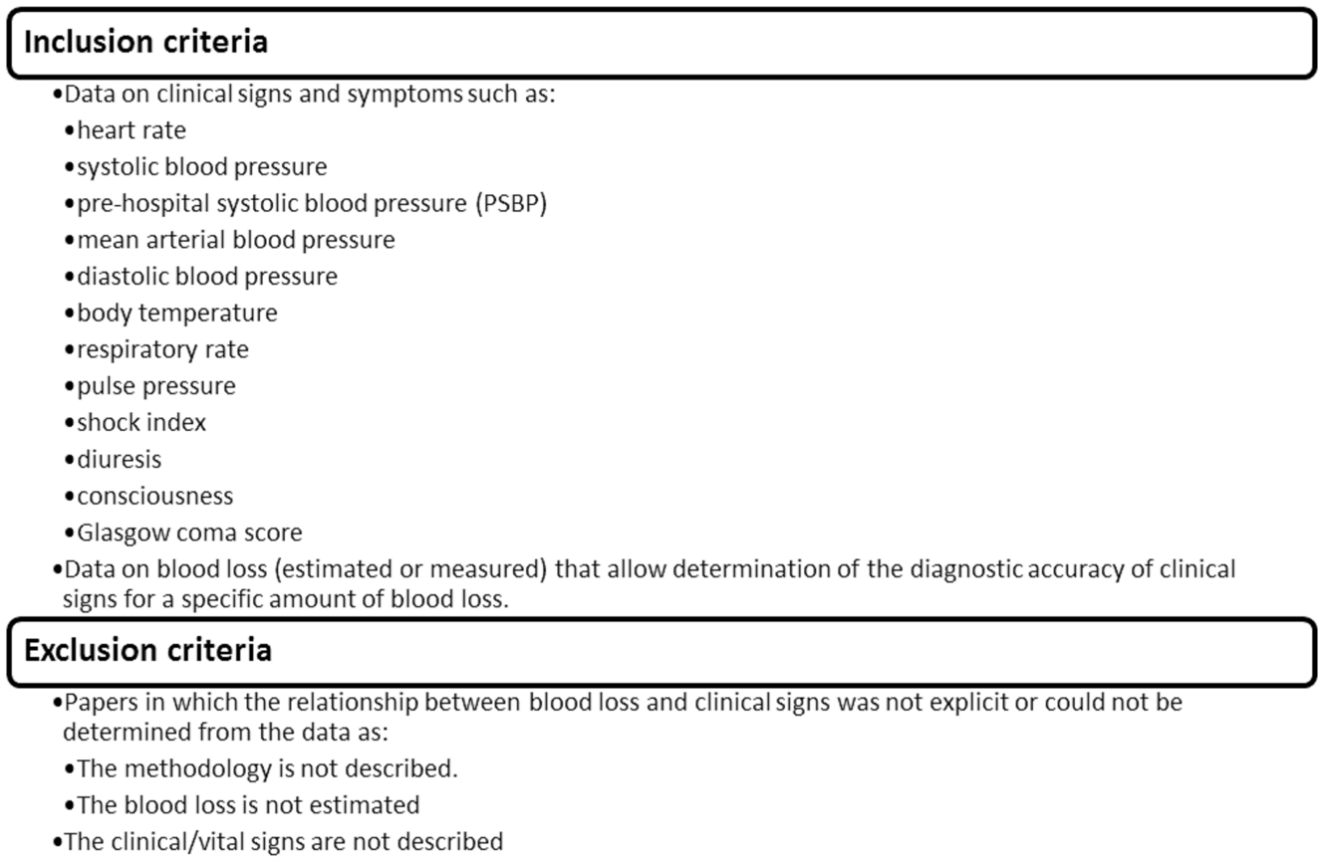
Inclusion and exclusion criteria.

We conducted a systematic review of the literature following the Meta-Analysis Of Observational Studies in Epidemiology (MOOSE) guidelines and the Preferred Reporting Items for Systematic reviews and Meta-Analyses (PRISMA) statement [12]. The primary focus of this systematic review is postpartum haemorrhage and other pregnancy-related bleeding. We included studies presenting data on the clinical signs and symptoms in relation to blood loss estimations in order to assess the diagnostic accuracy of clinical signs for a specific amount of blood loss. Due to the anticipated paucity of data from obstetric populations, we also included studies conducted in other populations. Papers in which the relationship between blood loss and clinical signs was not clear or could not be determined were excluded (Figure 1).

An electronic search was conducted in February 2012 using international study databases including Medline, EMBASE, Lilacs, Scielo, ISI and Google Scholar. We did not restrict the search strategy to exclude papers published in other languages, or studies of specific populations or study design. The search strategies used in each database are available in Appendix S1. All citations identified through the electronic search had their titles and/or abstracts examined. All potentially relevant papers were retrieved and assessed in detail. All manuscripts that were fully retrieved had their reference lists screened to identify other potentially relevant papers. The final set of papers to be included in the review was determined by consensus by two reviewers (RCP and JPS).

A pre-designed form was used independently by two reviewers (RCP and JPS) to conduct study eligibility assessment, critical appraisal and data collection. Data on the following variables were collected: type of study, population, blood loss assessment method, clinical signs data (i.e. heart rate, systolic blood pressure, pre-hospital systolic blood pressure (PSBP), mean arterial blood pressure, diastolic blood pressure, body temperature, respiratory rate, pulse pressure, shock index, diuresis, Glasgow coma score), clinical-sign assessment method and statistical method used. These reviewers assessed the methodological quality of studies independently using the checklist of essential items described in the STROBE (Strengthening the Reporting of Observational studies in Epidemiology) [13] statement and the general guidelines of MOOSE.

The included studies were classified into three categories, according to the mode of blood loss estimation: direct measurement (i.e. using drapes, drains, suctions, or visual estimation), indirect measurement (e.g. weighing sponges, hemodynamic monitoring, blood loss simulation or proxies) and simulation of blood loss in healthy subjects. In each study, the presence of an association between blood loss and the occurrence of changes in vital signs was determined and classified as present or absent. Another analysis was performed using population categories, according to the effect condition (pregnancy related study population, trauma population and healthy population).

The Microsoft Excel software was used in the tabulation and analysis of the abstracted data. Since a meta-analysis would not be appropriate due to the variation in study designs, we were only able to perform a qualitative analysis of the correlation between clinical signs and symptoms and the estimation of blood loss.

## Results

**Figure 2:**
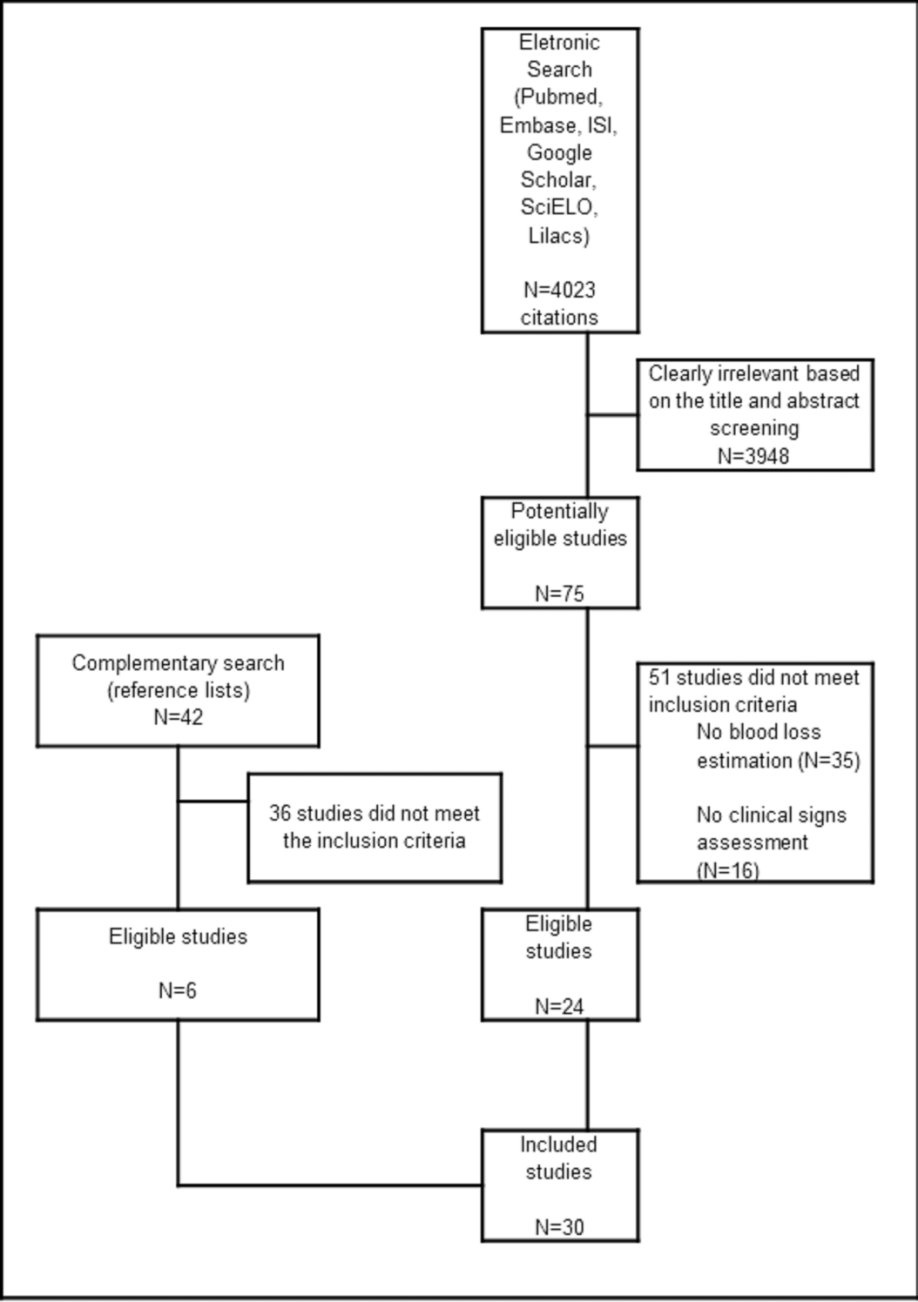
Flow diagram of identification and retrieval of examined studies.

A total of 4023 citations was identified by the electronic search and 75 manuscripts were retrieved for full-text assessment. Review of the reference lists of the selected articles resulted in the identification of 6 additional studies. In total, 30 studies were included in the systematic review (Figure 2).

Only five studies were performed in women with pregnancy-related haemorrhage: one study evaluated women with PPH and four included women with ectopic pregnancy (Table 1). The majority of the studies (19 out of 30) was related to haemorrhage due to trauma. Five studies were experimental and consisted of simulation of blood loss through the use of Low-Body Negative Pressure or Tilt-test in healthy male study subjects. In eight studies, methods enabling direct blood loss estimation were used. Indirect methods and simulation methods were used in 17 and 5 studies, respectively. Most studies were conducted in the United States of America (26 out of 30) and none were conducted in developing countries. The studies’ sample sizes ranged from 6 to 20 in the simulation group, from 116 to 199,657 participants (median  = 404) in the indirect blood loss estimation group and from 26 to 280 in the direct measurement group.

**Table 1:** Characteristics of included studies.

Study	Population	Blood loss estimation method		Country	Participants
*Direct Blood Loss estimation*					
**Birkhahn (2002)** [33]	Ectopic Pregnancy	Qualitative	Ruptured ectopic pregnancies	USA	280
**Birkhahn (2003)** [34]	Ectopic Pregnancy	Quantitative	Ruptured EP and visual estimation of hemoperitoneum	USA	52
**Hick (2001)** [27]	Ectopic Pregnancy	Quantitative	Aspiration of the abdominal cavity	USA	50
**Jaramillo (2010)** [35]	Ectopic Pregnancy	VEBL	Visual estimation of hemoperitoneum	USA	65
**Robson (1989)** [28]	Postpartum	VEBL	Clinically Estimation >500 ml	England	40
**Birkhahn (2005)** [31]	Healthy Subjects	Quantitative	Blood donation of 450 ml	USA	46
**Baron (2004)** [36]	Trauma	Qualitative	Chest tube drainage, intraoperative blood loss, and radiographic evidence of bleeding	USA	108
**Scalea (1990)** [37]	Trauma	Quantitative	Drainage of cavity OR Ht modification	USA	26
*Indirect Blood Loss estimation*					
**Brasel (2007)** [22]	Trauma	Proxies	Intervention to stop bleeding	USA	10,825
**Bruns (2007)** [38]	Trauma	Proxies	Intervention to stop bleeding	USA	404
**Bruns (2008)** [39]	Trauma	Proxies	Requiring blood transfusion	USA	16,365
**Cancio (2008)** [40]	Trauma	Proxies	Requiring blood transfusion	USA	536
**Chen (2007)** [41]	Trauma	Proxies	Requiring blood transfusion and bleeding trauma	USA	492
**Chen (2008)** [42]	Trauma	Proxies	Requiring blood transfusion and bleeding trauma	USA	358
**Edelman (2007)** [43]	Trauma	Proxies	Requiring blood transfusion	USA	2,071
**Guly (2011)** [19]	Trauma	Proxies	Clinical estimation based on bloodloss in specific injuries	England/Wales	199,657
**Hagiwara (2010)** [44]	Trauma	Proxies	Requiring blood transfusion > = 4 un	Japan;	261
**Luna (1989)** [45]	Trauma	Proxies	Requiring blood transfusion > = 5 un	USA	116
**McLaughlin (2009)** [46]	Trauma	Proxies	Requiring blood transfusion	USA	302
**Opreanu (2010)** [47]	Trauma	Proxies	Intervention to stop bleeding	USA	388
**Parks (2006)** [48]	Trauma	Proxies	Base deficit estimation as a marker of shock	USA	117,686
**Vandromme (2010)** [49]	Trauma	Proxies	Requiring Blood Transfusion >6 Un/24 h	USA	787
**Vandromme (2011a)** [30]	Trauma	Proxies	Requiring Blood Transfusion >10 Un/24 h	USA	8,111
**Vandromme (2011b)** [50]	Trauma	Proxies	Requiring blood transfusion >10 un/24 h	USA	514
**Zarzaur (2008)** [51]	Trauma	Proxies	Requiring blood transfusion >4 un/48 h	USA	16,077
*Simulation*					
**Convertino (2006)** [52]	Healthy Subjects	Simulation	LBNP	USA	10
**Convertino (2009)** [53]	Healthy Subjects	Simulation	LBNP	USA	10
**Rickards (2008)** [54]	Healthy Subjects	Simulation	LBNP	USA	12
**Secher (1984)** [55]	Healthy Subjects	Simulation	Tilt-test	Denmark	6
**Ward (2010)** [56]	Healthy Subjects	Simulation	LBNP	USA	20

VEBL – Visual estimation of blood loss; LBNP – Low body negative pressure.

An overall assessment of the methodological quality of the included studies is presented in the Table 2. Nine studies were considered of high quality. Detailed description of the study methods were lacking in most of the studies included in this review. For instance, 21 studies did not describe or provide sufficient detail of the study population, the health status of the population or the inclusion criteria.

**Table 2:** Critical appraisal of included studies.

Study	Type of study	Quality
**Birkhahn (2002)**	Diagnostic Test Accuracy	High
**Convertino (2009)**	Experimental (simulation)	High
**Birkhahn (2005)**	Prospective Cohort	High
**McLaughlin (2009)**	Prospective Cohort	High
**Chen (2007)**	Cross-Sectional	High
**Edelman (2007)**	Cross-Sectional	High
**Guly (2011)**	Cross-Sectional	High
**Hagiwara (2010)**	Cross-Sectional	High
**Vandromme (2010)**	Cross-Sectional	High
**Birkhahn (2003)**	Diagnostic Test Accuracy	Moderate
**Bruns (2007)**	Diagnostic Test Accuracy	Moderate
**Opreanu (2010)**	Diagnostic Test Accuracy	Moderate
**Convertino (2006)**	Experimental (simulation)	Moderate
**Rickards (2008)**	Experimental (simulation)	Moderate
**Ward (2010)**	Experimental (simulation)	Moderate
**Jaramillo (2010)**	Prospective Cohort	Moderate
**Luna (1989)**	Prospective Cohort	Moderate
**Robson (1989)**	Prospective Cohort	Moderate
**Vandromme (2011a)**	Prospective Cohort	Moderate
**HICK JL (2001)**	Retrospective Cohort	Moderate
**Vandromme (2011b)**	Retrospective Cohort	Moderate
**Baron (2004)**	Cross-Sectional	Moderate
**Brasel (2007)**	Cross-Sectional	Moderate
**Cancio (2008)**	Cross-Sectional	Moderate
**Parks (2006)**	Cross-Sectional	Moderate
**Zarzaur (2008)**	Cross-Sectional	Moderate
**Secher (1984)**	Experimental (simulation)	Low
**Scalea (1990)**	Prospective Cohort	Low
**Bruns (2008)**	Cross-Sectional	Low
**Chen (2008)**	Cross-Sectional	Low

The majority of studies did not provide information regarding the method of assessment of clinical signs. Of the 11 studies in which this information is available, only one performed the clinical evaluation using manual devices and ten performed such evaluations with automatic devices.

Table 3 summarizes the findings related to the association between clinical signs and blood loss. Heart rate, systolic blood pressure and the shock index were the clinical signs or clinical-sign derivatives most frequently studied. An association between blood loss and heart rate changes was observed in 22 out of 24 studies, and an association between blood loss and systolic blood pressure was observed in 17 out of 23 studies. One study showed an association between pre-hospital systolic blood pressure changes (i.e. measurement taken before reaching the hospital) and blood loss.

**Table 3:** Association between blood loss and changes in vital signs.

	Study	HR	SBP	SI	PSBP	MAP	DBP	PP	BT	RR
**Pregnancy related population**	Birkhahn (2002)	**•**	**•**	**•**		**•**				
	Birkhahn (2003)	**•**	**○**	**•**						
	HICK JL (2001)	**○**	**○**							
	Jaramillo (2010)			**•**						
	Robson (1989)	**•**	**○**				**○**			
**Healthy subjects**	Birkhahn (2005)	**•**	**•**	**•**			**○**			
	Convertino (2006)	**•**	**•**			**○**	**•**	**•**		
	Convertino (2009)	**•**	**•**			**•**	**•**			
	Rickards (2008)	**•**	**•**					**•**		**○**
	Secher (1984)	**•**				**•**		**○**		
	Ward (2010)	**•**				**•**				
**Trauma patients**	Baron (2004)	**•**	**○**	**•**		**○**				
	Scalea (1990)	**•**	* **•**					* **•**		
	Brasel (2007)	**•**								
	Bruns (2007)	**•**	**•**							
	Bruns (2008)		**○**		**○**					
	Cancio (2008)	**•**	**•**	**•**			**•**	**○**		**○**
	Chen (2007)	**•**	**•**	**•**			**○**	**•**		**○**
	Chen (2008)	**•**								
	Edelman (2007)		**•**							
	Guly (2011)	**•**	**•**							**○**
	Hagiwara (2010)	**•**	**•**	**•**			**•**		**•**	
	Luna (1989)	**○**	**○**		**○**					
	McLaughlin (2009)	**•**	**•**				**•**		**•**	
	Opreanu (2010)	**•**	**•**							
	Parks (2006)		**•**							
	Vandromme (2010)		**•**		**•**					
	Vandromme (2011a)			**•**						
	Vandromme (2011b)	**•**	**•**							
	Zarzaur (2008)	**•**	**•**	**•**						

BLE - Blood loss estimation; HR - Heart Rate; SBP - Systolic Bood Pressure; SI - Shock Index; PSBP - Prehospital Systolic Blood Pressure; MAP - Mean Arterial Pressure; DBP - Diastolic Blood Pressure; PP - pulse pressure; BT - Body Temperature; RR - respiratory rate;

•there is an association between blood loss and changes in the vital sign; ○: there is no association between blood loss and changes in the vital sign;

* •: no specification if systolic or diastolic.

A statistically significant association was found in all 10 papers reporting on the relationship of shock index (SI) and blood loss. Fewer studies evaluated the relationship between blood loss and other clinical signs: mean arterial pressure (4 out of 6 found an association), diastolic pressure (5/8), pulse pressure (4/6) and body temperature (2/2). Respiratory rate, diuresis and Glasgow coma scale were not associated with blood loss. In the subgroup of studies including only women with pregnancy-related blood loss, associations between blood loss and the shock index, heart rate and systolic blood pressure were found.

Several approaches were used to assess the relationship between clinical signs and blood loss in the included studies (Table 4). Seven studies used Received Operator Characteristic Curves to determine the accuracy of clinical signs in predicting blood loss. In those studies, the Area Under Curve (AUC) ranged from 0.56 to 0.74 for heart rate, from 0.56 to 0.79 for SBP and from 0.77 to 0.84 for shock index (Table 5). Seven studies (Table 6) provided information on the relationship between clinical signs and mortality. Of those, one study found HR and SI associated with higher mortality, and all of them found that low SBP was associated with higher mortality.

**Table 4:** Clinical signs assessment and blood loss estimation method.

Study	Clinical signs assessment	Blood loss estimation method
**Hick (2001)**	Automatic	Aspiration of the abdominal cavity
**Robson (1989)**	Automatic	Clinically Estimation >500 ml
**Convertino (2006)**	Automatic	LBNP
**Convertino (2009)**	Automatic	LBNP
**Rickards (2008)**	Automatic	LBNP
**Ward (2010)**	Automatic	LBNP
**Chen (2007)**	Automatic	Requiring blood transfusion and bleeding trauma
**Chen (2008)**	Automatic	Requiring blood transfusion and bleeding trauma
**Birkhahn (2002)**	Automatic	Ruptured ectopic pregnancies
**Secher (1984)**	Automatic	Tilt-test
**Birkhahn (2005)**	Manual	Blood donation of 450 ml
**Parks (2006)**	Non Available	Base deficit estimation as a marker of shock
**Baron (2004)**	Non Available	Chest tube drainage, intraoperative blood loss, and radiographic evidence of bleeding
**Guly (2011)**	Non Available	Clinical estimation based on blood loss in specific injuries
**Scalea (1990)**	Non Available	Drainage of cavity **OR** Hct modification
**Brasel (2007)**	Non Available	Intervention to stop bleeding
**Bruns (2007)**	Non Available	Intervention to stop bleeding
**Opreanu (2010)**	Non Available	Intervention to stop bleeding
**Bruns (2008)**	Non Available	Requiring blood transfusion
**Cancio (2008)**	Non Available	Requiring blood transfusion
**Edelman (2007)**	Non Available	Requiring blood transfusion
**McLaughlin (2009)**	Non Available	Requiring blood transfusion
**Vandromme (2011a)**	Non Available	Requiring Blood Transfusion >10 Un/24 h
**Vandromme (2011b)**	Non Available	Requiring blood transfusion >10 un/24 h
**Hagiwara (2010)**	Non Available	Requiring blood transfusion > = 4 un
**Luna (1989)**	Non Available	Requiring blood transfusion > = 5 un
**Zarzaur (2008)**	Non Available	Requiring blood transfusion >4 un/48 h
**Vandromme (2010)**	Non Available	Requiring Blood Transfusion >6 Un/24 h
**Birkhahn (2003)**	Non Available	Ruptured EP and visual estimation of hemoperitoneum
**Jaramillo (2010)**	Non Available	Visual estimation of hemoperitoneum

VEBL – Visual estimation of blood loss; LBNP – Low body negative pressure.

**Table 5:** Clinical signs statistical analysis.

Study	Statistic	HR	SBP	PSBP	MAP	DPBP	PP	SI	cuttoff SI
**Birkhahn (2002)**	AUC	0.74	0.7		0.63			0.84	0.85
**Brasel (2007)**	AUC	0.56–0.59							
**Chen (2007)**	AUC	0.66	0.71			0.58	0.73	0.77	
**Opreanu (2010)**	AUC	0.59	0.56						
**Vandromme (2010)**	AUC		0.6	0.61					
**Vandromme (2011b)**	AUC	0.65	0.79						
**Zarzaur (2008)**	AUC	0.73	0.71					0.78	0.83
*Range*		*0.56–0.74*	*0.56–0.79*	*0.61*	*0.63*	*0.58*	*0.73*	*0.77–0.84*	
**Parks (2006)**	Pearson coef		0.28						
**Scalea (1990)**	Pearson coef	0.199	0.004*				0.005		
**Birkhahn (2003)**	Pearson coef	0.50	−0.34					0.69	0.7
**Hick (2001)**	R^2^	0.04	0.1						
**Jaramillo (2010)**	R^2^							0.57	
*Range*		*0.04–0.199*	−*0.34–0.28*				*0.005*	*0.57–0.69*	

AUC –Area under curve; HR - Heart Rate; SBP - Systolic Bood Pressure; SI - Shock Index; PSBP - Prehospital Systolic Blood Pressure; MAP - Mean Arterial Pressure; DPBP - Diastolic Blood Pressure; PP - pulse pressure; BT - Body Temperature; RR - respiratory rate; *BP – no specification if systolic or diastolic.

**Table 6:** Clinical signs associated to mortality in included studies.

Study	Mortality
**Zarzaur (2008)**	HR/SBP/SI
**Bruns (2008)**	SBP
**Cancio (2008)**	SBP
**Edelman (2007)**	SBP
**Luna (1989)**	SBP
**Parks (2006)**	SBP
**Vandromme (2010)**	SBP
Victorino (2003)	SBP+TACHYCARDIA

## Discussion

This systematic review identified 30 scientific papers reporting on the relationship between blood loss and clinical signs. Overall, these studies found a substantial variability in the relationship of blood loss and clinical signs, making it difficult to establish specific cut-off points for clinical signs that could be used as triggers for clinical interventions. However, the shock index seems to be a promising indicator of the severity of blood loss.

To the best of our knowledge this is the first systematic review of studies assessing the relationship of blood loss and clinical signs in the context of pregnancy and childbirth. This review was conducted following the most recent methodological guidelines for reviews of this kind and did not have any restrictions in terms of language and source of data. Nevertheless, some limitations need to be noted. First, this review intends to inform decisions concerning pregnancy-related haemorrhage. Due to paucity of data on obstetric populations, most of the studies included in this review come from non-obstetric populations. This is a relevant issue considering that women experience substantial physiological changes during pregnancy (e.g. increase in the maternal blood volume and cardiac output, reduction of cardiovascular reserve) [14], [15]. A second issue that needs to be considered is that most of the women experiencing severe complications related to postpartum haemorrhage are in developing countries. Anemia during pregnancy due to iron deficiency or other factors (e.g. malaria) affects up to 55% of pregnant women from low and middle income countries compared with around 20% or less from high income countries [16], [17]. Anemia may impair the physiological response to blood loss and worsen maternal prognosis. So, the evidence generated by this systematic review needs to be considered in the context of indirectness due to differences in population and setting.

Another limitation is that different methods of blood loss measurement were used across the studies. Ideally, the use of direct methods would be desirable in this kind of research. The majority of studies we reviewed used proxies to define the severity of blood loss, which may introduce a considerable bias in the analysis. A proxy measure for evaluating blood loss based on red-cell transfusion is influenced by other factors, including provider and patient behaviors and attitudes towards transfusion, as well as the availability of blood at some hospitals, thus altering the blood loss estimation. In addition, only few studies described the methods used to assess clinical signs. The use of different techniques to obtain data on blood pressure, heart hate, respiratory rate, pulse pressure and other clinical data may increase the heterogeneity of results. Furthermore, clinical signs may also be affected by other factors such as the use of caffeine and alcohol or even by labour per se which can enhance heart rate, mean arterial pressure and cardiac output during contractions [18]. The experimental studies controlled for such factors, but we found no evidence of controlling for this potential bias in the observational studies reviewed.

In spite of these limitations, the studies included in this systematic review did show an association between blood loss and changes in clinical signs in the non-obstetric population. However, there is substantial variation in the response of clinical signs to blood loss, which limits their applicability in diagnosing haemorrhage or guiding its management. Guly and colleagues [19] found an association between high heart rate, low systolic blood pressure and the amount of blood loss in seriously injured patients but not to the degree suggested by the classification of the American College of Surgeons in the Advanced Trauma Life Support (ATLS) program [20]. Other authors have found that tachycardia (defined as a HR over 90 bpm) is neither sensitive nor specific for the diagnose of hypotension and amount of blood loss [21]. For Brasel et al. [22] tachycardia (defined as a pulse greater than 100 bpm) had poor sensitivity and specificity (less than 37% and 79% respectively) in identifying substantial blood loss. Although the ATLS classification system for hypovolemic shock is widely used, the proposed cut-off values for clinical signs have been challenged. SBP values that are higher than what is usually considered as “hypotension” have been associated with increased morbidity and mortality [19], [23]. It has been suggested that hypotension should be redefined using a higher cut-off blood pressure than actually used in the general population [23]–[26].

The physiological changes in the cardiovascular system during pregnancy and postpartum may hinder early recognition of hypovolemia and delay treatment. In a first-trimester pregnancy population, the correlation between vital signs and the amount of blood in the peritoneal cavity was shown to be poor. Hick and colleagues [27] did not find any association of clinical signs and hemoperitoneum. The authors assume that if surgical decisions were made based on clinical signs, more than one third of patients might be treated inappropriately. Another study using an obstetric population in late pregnancy found similar data with no correlation between blood loss and blood pressure [28]. During late pregnancy and the postpartum period, physiological changes in the cardiovascular system are even more substantial. In the case of PPH, some variables have been suggested to improve clinical judgment for PPH treatment (e.g. clinical signs and symptoms, visual estimation of blood loss, and the blood loss rate) but none have been sufficiently tested. Some authors suggest changing the blood-loss based definition of PPH to a system of signs and symptoms of hypovolemia. A hypovolemic shock classification system was proposed using classes of hemorrhage correlating signs and symptoms to the amount of blood lost and to a fluid replacement procedure [8], [11], [29]. According to this classification, a compensated shock occurs with a blood loss of less than 1000 ml and no change or slight change in clinical signs. Substantial changes in heart rate and blood pressure would be seen after a blood loss of more than 1000 ml. Hypotension with significant tachycardia and rise in respiratory rates would occur after a loss of 25–35% of blood volume and profound shock occurs after a 40% blood loss. However, the use of clinical signs may lack accuracy in the assessment of hypotension and needs further testing in order to help guide the management of PPH.

Overall, our review findings suggest that blood loss is associated with changes in clinical signs but it is difficult to establish robust cut-offs that could guide the management of women with pregnancy-related haemorrhage. On the other hand, when it comes to a clinical sign derivative – the shock index – our review findings are more encouraging. The shock index is calculated as the heart rate divided by the systolic blood pressure and this simple calculation may transform unstable parameters into a more accurate predictor of hypovolemia. According to studies included in this review, the shock index may identify hypovolemia even in patients who otherwise would be considered with no hypotension [30], [31]. In addition, the shock index has been recently suggested as a tool to predict mortality due to hypovolemic shock in trauma patients. The use of the shock index in the early identification and assessment of bleeding is considered promising even in obstetric populations [32]. Birkhahn and colleagues [33] studied first-trimester pregnant women with abdominal pain and found that a shock index >0.85 was highly suggestive of the presence of hemoperitomeum due to ruptured ectopic pregnancy. This parameter was found to be a better predictor of bleeding than HR or SBP only [34]. Similar findings were obtained by other authors suggesting that shock index may be a good criteria for early diagnosis of haemorrhage [35].

### Conclusion

This systematic review found a substantial variability in the relationship between blood loss and clinical signs, making it very difficult to establish specific cut-off points for clinical signs that could be used as triggers of clinical interventions. However, the shock index was found to be an accurate indicator of compensatory changes in the cardiovascular system due to blood loss. Considering that most of the evidence included in this systematic review is derived from studies in non-obstetric populations, further studies on the use of the shock index in obstetric populations are needed.

## Supporting Information

Appendix S1 **Preliminary Search Strategy in Medline.**
(DOCX)

Checklist S1 **PRISMA Checklist.**
(DOC)
