# Correction: A Systematic Review of the Relationship between Blood Loss and Clinical Signs

**DOI:** 10.1371/annotation/4db90e4b-ae29-4931-9049-3ef5e5c9eeee

**Published:** 2013-06-18

**Authors:** Rodolfo Carvalho Pacagnella, João Paulo Souza, Jill Durocher, Pablo Perel, Jennifer Blum, Beverly Winikoff, Ahmet Metin Gülmezoglu

There was an error in affiliation 1 for author Rodolfo Carvalho Pacagnella. Affiliation 1 should be University of Campinas, Campinas, Brazil.

