# Supplementary material for: A Systematic Review of the Relationship between Blood Loss and Clinical Signs
Source: PLoS One. 2013 Mar 6;8(3):e57594. doi: 10.1371/journal.pone.0057594 (PMC3590203; doi:10.1371/journal.pone.0057594)
Supplement: Appendix S1 — Preliminary Search Strategy in Medline. (DOCX) [file pone.0057594.s001.docx]

**Appendix S1**

**Preliminary Search Strategy in Medline**

((Shock) OR (Blood Loss)) AND ((Diagnosis) OR (Classification)) AND ((clinical signs correlation) OR (Blood Pressure) OR (Heart Rate) OR (Respiratory rate) OR (consciousness) OR (temperature) OR (perfusion)) AND ((Obstetric) OR (Pregnancy) OR (trauma) OR (Hemoperitoneum) OR (Uterine) OR (Rupture) OR (Atony)) AND (Adult)
